# An Overview of α-Pyrones as Phytotoxins Produced by Plant Pathogen Fungi

**DOI:** 10.3390/molecules30132813

**Published:** 2025-06-30

**Authors:** Antonio Evidente

**Affiliations:** Institute of Biomolecular Chemistry, National Research Council (CNR), Via Campi Flegrei 34, 80078 Pozzuoli, Italy; evidente@unina.it

**Keywords:** crop diseases, phytopathogen fungi, phytotoxins, pyrones, chemical and biological characterization

## Abstract

Crop diseases negatively affect the quality and quantity of agricultural products, with significant economic and social consequences. These problems become emergencies in a world where the safe production of food for human health is becoming increasingly pressing. Microorganisms, including phytopathogenic fungi, are the main organisms responsible for these diseases, which cause devastating damage. Environmental pollution generated by human activities causes further significant reductions in agricultural production, as well as the expansion of metropolitan areas, and climate change. Phytotoxins produced by pathogenic fungi play a fundamental role in the induction of diseases by directly interfering with the physiological processes of agricultural plants. They are secondary metabolites that can belong to all the different classes of natural compounds, and their structures and biological activities have been extensively studied. These substances have often been shown to possess other interesting biological activities for potential applications both in agriculture and in other fields, such as biotechnology and medicine. This review focuses on phytotoxic α-pyrones produced by plant pathogenic fungi, describing in detail all their chemical and biological properties and, in some cases, the results of studies on their structure-activity relationship and on the potential practical applications in various sectors.

## 1. Introduction

Nature is an almost inexhaustible source of a myriad of compounds belonging to several different groups of organic substances, exhibiting specific biological activities [1]. Aromatic heterocycle compounds are about two-thirds of the total organic compounds that define the history, present, and future of modern drugs. Benzene rings in which one carbon (C) is replaced by heteroatoms, such as nitrogen (N), oxygen (O), and sulfur (S), generate different subgroups of heterocyclic compounds [2]. Pyrones are a class of heterocyclic six-membered lactones represented by two isomers, 2-pyrone and 4-pyrone. The number 2/4 is assigned on the basis of the position of the carbonyl group relative to the oxygen atom within the ring system. The 2-pyrone (more commonly known as α-pyrone) structure is found in nature as part of the coumarin ring system. 4-Pyrone (more commonly known as γ-pyrone) is found in some natural chemical compounds such as chromone, maltol, and kojic acid [3]. There is a growing interest in α-pyrones due to their structural diversity and their diverse biological activities, which include anti-inflammatory, cytotoxic, antibacterial, antioxidant, insecticidal, and antifungal activity [4,5,6,7]. α-Pyrones are not only produced by fungi, but also, although less frequently, by bacteria, by plants, and by lichens.

Some reviews have previously addressed this topic, but they only report in isolation, either chemical or biological characterization of the α-pyrones, and sometimes also cover a limited time range of publications. Azizian et al. (2024) [8] reported the chemical structures and biological activities of 268 natural α-pyrone derivatives, isolated from January 2016 to October 2023, as fungi (199), bacteria (34), and plant (35) metabolites. Successively, another review describes natural compounds containing a 2-pyrone moiety, emphasizing their role in biological activity, particularly with respect to potential therapeutic or antimicrobial agents [5]. Other authors confirmed the pharmacological properties and potential applicability of the 2-pyrones, focusing attention on their chemotherapeutic activities. The α-pyrones are reported as anti-HIV, anti-TB, and anti-cancer agents, and for their potential role against neurodegeneration, hypercholesterolemia, microbial infections, chronic obstructive lung disease, inflammation, antinociception, and immunomodulation [9]. Some authors reported the relation of the stereochemistry of the α-pyrones with their biological activity [10], while other ones selectively described those produced by fungi and with potential herbicidal activity [11]. Schäberle (2016) [7] specifically reported new insights into additional biosynthetic mechanisms of α-pyrones, while other authors specifically described the chemical and biological properties of natural diterpene pyrones [12].

Thus, the present review is the first one focused on only fungal pyrones with phytotoxic activity. The results discussed in the three different sections were obtained from Sci-Finder and chronologically reported. Furthermore, where reported for some phytotoxic *a*-pyrones, the results of structure–activity, mode-of-action studies, and future perspectives are also discussed.

## 2. α-Pyrones

### 2.1. α-Pyrones Produced from Fungi Pathogenic for Agrarian Plants

**Figure 1:**
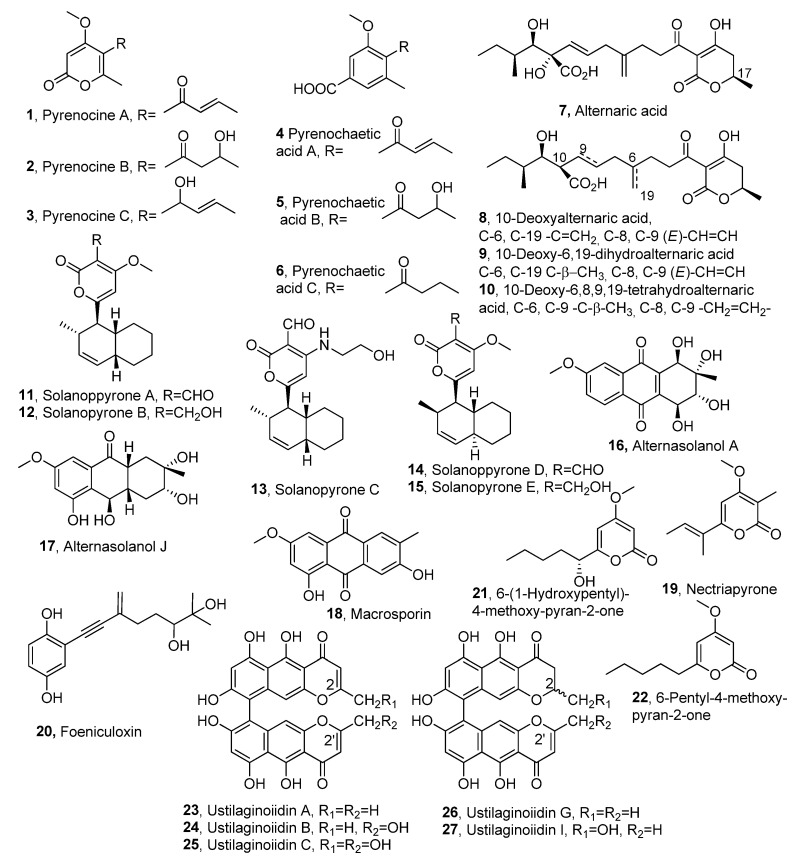

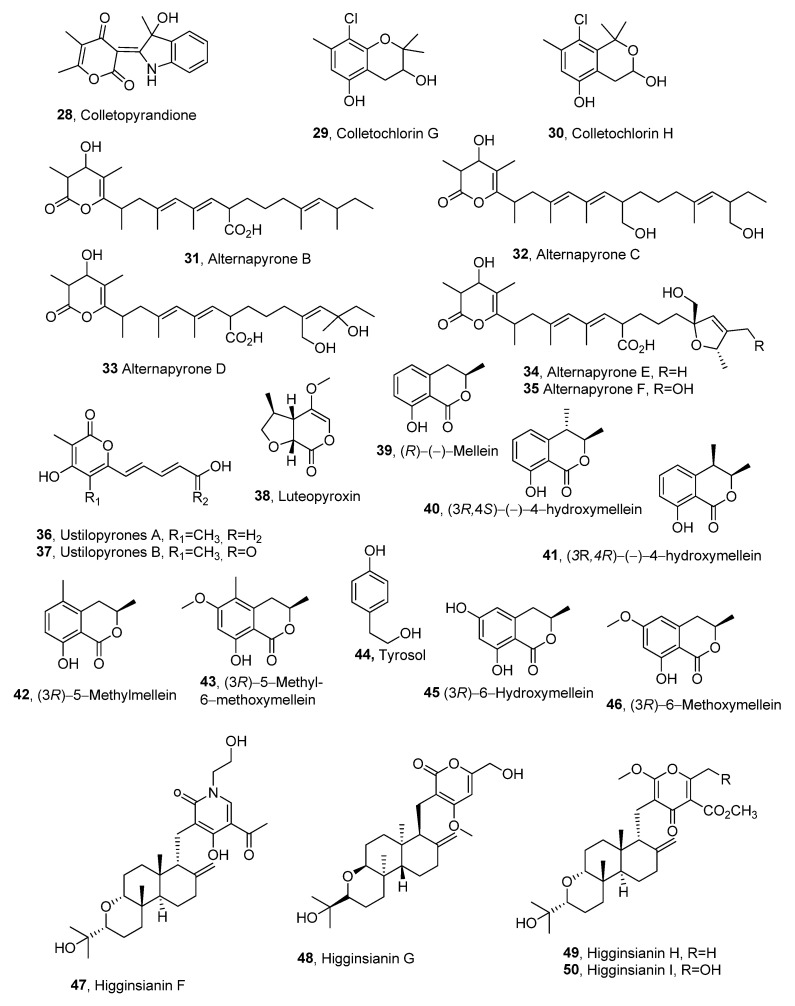
Structures of phytotoxic α-pyrones produced by fungal pathogens for agrarian plants.

Some differently substituted α-pyrones were produced in liquid culture by the fungus *Pyrenoclzaeta terrestris*, which is the causal agent of onion pink disease, attracting considerable attention in outbreaks in the land where onion cultivation is widespread. The fungus was also isolated from other plants such as rice and garlic. The first phytotoxins isolated were pyrenocines A and B (**1** and **2**, Figure 1, Table 1) [13]. Toxin **1** was also isolated from *Trichoderma citro-viride* and named citreopyrone [14]. The phytotoxic activity induced by compound **1** on onion root was more strong than that of compound **2** [13]. The first furane-derivative structure, assigned to both phytotoxins, was then revised as that of two α-pyrone derivatives by X-ray analysis of pyrenocine A and that of pyrenocine B by spectroscopic correlation [15]. Successively, from the same culture filtrates, pyrenocine C (**3**, Figure 1, Table 1) was also isolated and showed only a weak phytotoxicity [16]. When *P. terrestris* was grown on solid culture, it also produced three other phytotoxins, named pyrenochaetic acids A-C (**4**–**6**, Figure 1, Table 1), which are close to pyrenocines. The phytotoxicity of pyrenochaetic acids A-C was tested on the growth of onion and rice seedlings by the germination test using lettuce seed. Pyrenochaetic acid A (**4**) inhibited the root growth of onion and rice seedlings by 100% at 250 and at 5000 ppm, respectively, while its analogue **6** was less toxic. In the germination test on lettuce seeds, the three acids (**4**–**6**) exhibited equal inhibition at high concentrations, but promoted root elongation at low concentrations [17]. Successively, pyrenocin A (**1**) showed antibiotic activity against plants, fungi, and bacteria. In particular, it showed an effective dose for 50% inhibition (EDSO) of 4 kg/mL for elongation of onion seedlings, and 14, 20, 20, and 25 kg/mL for the germination of asexual spores of *Fusarium oxysporum* f. sp. *cepae*, *Fusarium solani* f. sp. *pisi*, *Mucor hiemalis,* and *Rhizopus stolonifer*, respectively. Compound 1 also inhibited linear mycelial growth of *P. terrestris* and *F. oxysporum* with an EDSO of 77 and 54 kg/mL, respectively. Furthermore, pirenocin A (**1**) showed biostatic rather than biocidal activity against all the bacteria used, with Gram-positive bacteria being more sensitive than Gram-negative bacteria. In fact, the EDOs observed for the growth inhibition of *Bacillus subtilis*, *Staphylococcus aureus,* and *Escherichia coli* were 30, 45, and 200 kg/mL, respectively, while *Pseudomonas aeruginosa* appeared to be resistant at the concentrations used. Pirenocins B and C (**2** and **3**) showed weak antibiotic activity in all the tests performed [18].

*Alternaria solani*, the causal agent of early blight disease of tomatoes and potatoes [44], produced different non-related toxins as alternaric acids, a5,6-dihydro-α-pyrones and solanapyrones [19]. Alternaric acid (**7**, Figure 1) induced, in the host plant, symptoms similar to those of the so-called host-specific toxins. This phytotoxin (**7**) was also shown to delay the occurrence of hypersensitive death of potato cells infected by an incompatible race of *Phytophthora infestans* [45]. Then the determination of the stereochemistry and the total synthesis of alternaric acid **7** were also reported [46]. Three other alternaric acid-related compounds, 10-deoxyalternaric acid, 10-deoxy-6,19-dihydroalternaric acid, and 10-deoxy-6,8,9,19-tetrahydroalternaric acid (**8**–**10**, Figure 1, Table 1), were isolated from the same fungus [20]. All the alternaric acids (**7**–**10**) were tested for their growth inhibition of tomato seedlings and showed a different rate of phytotoxicity on roots and hypocotyls. The results obtained suggested that their phytotoxicity depends on the oxidation levels of the alternaric acids, and that the exo-methylene group at C-6 and the hydroxy group at C-10 in alternaric acid **7** play an important role in the phytotoxic activity. Furthermore, the phytotoxic activity of the degradation and the synthetic segments of alternaric acid suggested that the side-chain moiety and the 3-acyl-4-hydroxy-5,6-dihydro-2-pyrone moiety play different roles in the phytotoxic activity [20]. Solanapyrones A-E (**11**–**15**, Figure 1, Table 1) were produced from another strain of *A. solani* [21,22,23]. Both solanapyrones A and D, and B and E were obtained in a diastereomeric ratio of 6:1 [19]. Solanapyrones A and C were also isolated from filtrates of stationary cultures of *Ascochyta rabiei*, the causal fungus of chickpea blight [47], which is the most important disease of chickpea in areas where the growing season coincides with cool and moist weather [48]. The disease caused severe losses around the Mediterranean Basin and in Pakistan, where a loss of up to 50% of the crop was observed. Compounds **11** and **12** showed synergistic activity when tested on potatoes, while on isolated cells of the leaflets of 10-day-old chickpea seedlings, their effect was additive. The solanapyrones share interesting structural similarities to several phytotoxins, such as betaenones A and B isolated from *Phoma betae*, a parasite of sugar beet [49], and stemphyloxin from *Stemphylrum botryosum,* the causal agent of leaf spot on tomatoes [50]. Compounds **11** and **12** could act as siderophores to disturb the non-metabolism of the host. However, solanapyrones probably do not act directly as iron chelators per se, although they may chelate iron or other essential metal ions upon ring opening of the pyrone or upon further biotransformation within the host [47]. Although the phytotoxicity of solanapyrone A has been reported many times, its role in pathogenicity has not been completely clarified. A genetic study was carried out on the *sol5* gene, which encodes Diels-Alderase, which catalyzes the final step of solanapyrone biosynthesis. Silencing of this gene in both *A. rabiei* and *A. solani* leads to the accumulation of prosolanapyrone II-diol, which is the immediate biosynthetic precursor of solanapyrones and is not toxic to plants. Instead, solanapyrone A showed high toxicity against *Arabidopsis thaliana*. Furthermore, pathogenicity tests showed that non-solanapyrone-producing mutants of both fungi retained the virulence of wild-type strains. These results suggested that solanapyrones were not required for the pathogenicity of either fungus [51]. The antibacterial activity of solanapyrones A-C was tested against various human pathogens, such as Gram-positive, *B. subtilis*, *Bacillus megaterium*, *Clostridium perfringens*, *Micrococcus tetragenus*, MRSA (Methicillin-resistant Staphylococcus), and Gram-negative, *E. coli*, using streptomycin, acheomycin, and ampicillin as positive controls. Solanapyrone A (**4**) (MIC (Minimal Inhibitory Concentration) of 12.5 μg/mL) showed the same activity as ampicillin (MIC of 12.5 μg/mL) and better than that of streptomycin (MIC of 100 μg/mL) against *B. subtilis*. Solanapyrone B (**5**) showed better activity than ampicillin (MIC of 50 μg/mL) against *B. megaterium*. None of the tested compounds showed significant inhibition of the growth of *E. coli* and MRSA [24].

Altersolanols A and J, macrosporin (**16**–**18**, Figure 1, Table 1), three octaketides anthracenones, nectriapyrone (**19**, Figure 1, Table 1), and a pentaketide monoterpenoid, were isolated from *Diaporthe angelicae* (anamorph *Phomopsis foeniculi*), which is the causal agent of a heavy disease in fennel (*Foeniculum vulgare*) in Bulgaria [25]. Nectriapyrone was also named as a pestalopyrone when it was previously produced from an unidentified fungus isolated from the Indo-Pacific sponge *Stylotella* sp. [32,52]. The symptoms induced in the host plant by *D. angelicae* were umbel browning and stem necrosis [25]. The dried fennel seeds are used in phytotherapy and the pharmaceutical [53] and alimentary industries. When assayed using a leaf puncture bioassay on detached tomato leaves, nectriapyrone and altersolanols A and J showed a modulated phytotoxicity, while macrosporin was not toxic. Altersolanol A was the most phytotoxic compound [52]. The same pathogen fungus, but a strain isolated from diseased fennel near Florence (Italy), produced foeniculoxin (**20**, Figure 1), a phytotoxic geranylhydroquinone [28]. Foeniculoxin (**20**), at 3.6 10^−3^ M, reduced root growth of the germinant seeds of both fennel and tomato while inducing necrosis on tobacco leaves and wilting and/or spots on the leaves of tomato cuttings [28]. Successively, from the same fungal culture filtrates, two phytotoxic exopolysaccharides (EPSs), namely a galactan with the known structure [-->6)-beta-D-Galf-(1-->5)-beta-D-Galf-(1-->5)-beta-D-Galf-(1-->]n and a branched mannan, were isolated too. The branched mannan consists of a backbone of alpha-(1-->6)-linked mannopyranose units. Almost all of these are branched at the 2 position, with arms containing 2- and 3-linked mannopyranose units. The crude polysaccharide and the galactan and mannan showed phytotoxic activity, i.e., chlorosis, necrosis, and/or wilting, on fennel and two non-host plants, tobacco and tomato [54]. Furthermore, altersolanol A (**16**) exhibited in vitro cytotoxicity activity against 34 human cancer cell lines with a mean IC_50_ (IC_70_) values of 0.005 μg ml^−1^ (0.024 μg ml^−1^) respectively and inhibited kinase inducing cell death by apoptosis through the cleavage by Caspase-3 and -9 and by decreased anti-apoptotic protein expression [26]. In addition, macrosporin showed antifungal activity against *Colletotrichum musae*, *Colletotrichum gloeosporioides*, *Fusarium graminearum*, *Penicillium italicum*, *Fusarium oxysporum* f. sp. *lycopersici,* and *Rhizoctonia solani* at different levels. Noteworthy activity, compared to that of carbendazim used as a positive control, was shown by compound **18** towards *Fusarium graminearum* [27].

Pestalopyrone (=nectriapyrone, **19**), a pentaketide already known as a minor toxin produced by *Pestalotiopsis oenotherae* [55], was also isolated from *Pestalotiopsis guepinii*. [56]. *P. guepinii* is the fungal causal agent of the so-called ‘twig blight’, one of the most serious diseases in hazelnuts (*Corylus avellana* L.) in Turkey, which causes severe yield losses [56]. *P. guepinii* was also isolated from walnut (*Juglans* spp.) and gum mastic tree (*Pistacia lentiscus* var. Chia) [56]. When the fungus was grown on a different culture medium besides nectriapyrone, two other phytotoxic pyrones were isolated from *P. guepenii* and identified as 6-(1-hydroxypentyl)-4-methoxy- and 6-pentyl-4-methoxy-pyran-2-one (**21** and **22**, Figure 1, Table 1) [33]. Tested by puncture on leaves of a number of plant species (*Convolvulus arvensis*, *Mercurialis annua*, *Chenopodium album*, and *Ailanthus altissima*), compound **22** showed high phytotoxicity, inducing large necrosis on the leaves of all the species tested, while compound **21** showed a similar toxicity to α-pyrone **22**, although it did not cause necrosis on the leaves of *C. arvensis.* This difference was probably due to a lower sensitivity of the latter plant. These results suggested that the functionalities of the *n*-pentyl side chain are important for the activity. On *Lemna minor* L., compound **22** appeared to be the most toxic compound, causing the complete desiccation of the plantlets, fumonisin B1, which is a powerful phytotoxin used for comparison, while pestalopyrone (**19**) proved to be not toxic [33]. When isolated from *Cosmosporella* sp., an endophytic fungus of *Vinca minor*, pestalopyrone (**19**) showed a selective inhibition of Gram-positive bacteria, such as methicillin-sensitive and methicillin-resistant *S. aureus* with MIC and MBC (Minimum Bactericidal Concentration) values ranging from 125 to 62.5 µg mL^−1^ against MSSA and MRSA strains [31].

Five main bis-naphtho-γ-pyrones, namely ustilaginoidins A, B, C, G, and I (**23**–**27**, Figure 1, Table 1), were isolated from the rice false smut balls (FSBs) infected by *Villosiclava virens* in rice spikelets on panicles. Rice false smut has become an increasingly serious fungal disease in rice (*Oryza sativa* L.) production worldwide. The contents of five ustilaginoidins (**23**–**27**) in rice FSBs at early, middle, and late maturation stages were determined by HPLC analysis. The results showed that the highest levels of ustilaginoidins were found in rice FSBs at the late stage, followed by those at the intermediate stage. The contents of ustilaginoidins A (**23**) and G (**25**) were relatively high at the early stage, while the contents of ustilaginoidins B, C, and I (**24**, **25,** and **27**), having hydroxymethyl groups at C-2 or C-2′, were relatively high at the late stage [34]. Ustilaginoidin A (**23**) also inhibited ATP synthesis in mitochondria by uncoupling oxidative phosphorylation and depressing state-3 respiration of mitochondria [35].

Colletopyrandione, colletochlorins G and H, a tetrasubstituted chroman, and a tetrasubstituted isocroman-3,5-diol (**28**–**30**, Figure 1, Table 1), were isolated from the culture filtrates of the fungus *Colletotrichum higginsianum* together with 4-chloroorcinol, colletopyrone, and colletochlorins E and F [36]. *C. higginsiunum*, belonging to the *Colletotrichum destructivum* species complex [57], causes anthracnose leaf spot disease of several Brassicaceae crop species. Previously, from the mycelium of the same fungus were obtained colletochlorins A and B, which showed promising anticancer activity [58]. Assayed in several biological systems, colletopyrandione showed a modest phytotoxic activity, associated with a complete lack of toxicity towards off-target organisms [55]. Alternapyrones B-F (**31**–**35**, Figure 1, Table 1), which are five new α-pyrone polyketides, were produced by the fungal wheat pathogen *Parastagonospora nodorum*, whose biosynthetic gene cluster, which was significantly upregulated during plant infection, was heterologously reconstructed in *Aspergillus nidulans*. Compounds **34** and **35**, which contain a highly substituted dihydrofuran moiety, showed phytotoxic activity on wheat seed germination. Furthermore, only three enzymes, one highly reducing polyketide synthase and two multifunctional P450 oxygenases, were found to be necessary to synthesize the structurally complex α-pyrone [37].

Ustilopyrones A and B (**35** and **36**, Figure 1, Table 1), two sorbicillinoid-related pyrones, ustisorbicillinols A-F, and six sorbicillinoids were isolated together with nine known cogeners from *Ustilaginoidea virens*, the causal agent of rice false smut [38]. Phytotoxicity assays showed that the major sorbicillinoids bisvertinolone [59], demethyltrichodimerol [30], trichodimerol, and bislongiquinolide (also named trichotetronine) [29,60] showed strong inhibition against the radicle and germ elongation of rice and lettuce seeds, with bisverticolone being the strongest inhibitor. Dihydrotrichodimer ether A [41], and bisvertinolone, demethyltrichodimerol, trichodimerol showed moderate cytotoxicities against the tested cell lines with IC_50s_ of 8.83–74.7 µM, while ustisorbicillinol B, dihydrotrichodimer ether A, oxosorbicillinol [61], bisvertinolone, and demethyltrichodimerol were active against the tested bacteria (MICs (Minimal Inhibitory Concentrations) of 4∼128µg/mL). Furthermore, oxosorbicillinol bisvertinolone, demethyltrichodimerol displayed moderate antifungal activity [41].

Different phytotoxic metabolites were isolated from the organic extract of *Neofusicoccum luteum*, *Neofusicoccum australe*, and *Neofusicoccum parvum*, causal agents of Botryosphaeria dieback in Australia. *N. luteum* produced luteopyroxin (*R*)-(−)-mellein (1), and (3*R*,4*S*)-(−)- and (3*R*,4*R*)-(−)-4-hydroxymellein, (**38**–**41**, Figure 1, Table 1), a disubstituted furo-α-pyrone, a hexasubstituted anthraquinone, a trisubstituted oxepi-2(7*H*)-one, neoanthraquinone, luteoxepinone, (±)-nigrosporione, and tyrosol. The three melleins (**38**–**41**) and tyrosol were also produced by *N. parvum*, while *N. australe* produced (*R*)-(−)-mellein (**39**), neoanthraquinone, tyrosol, and *p*-cresol. When assayed on grapevine leaves, neoanthraquinone showed the highest toxic effect, causing severe shriveling and withering. Luteopyroxin (**38**), nigrosporione, and luteoxepinone also showed different degrees of toxicity, while p-cresol displayed low phytotoxicity [39]. Mellein and its two 4-hydroxy analogues (**38**–**41**) showed other interesting biological activities, including antibacterial activity against MRSA strains, larvicidal activity against *Aedes aegypti,* and antifungal activity towards several pathogens of agrarian plants, as extensively reported by the author of this review [40].

Nectriapyrone (**19**), (3*R*)-5-methylmellein, (3*R*)-5-methyl-6-methoxymellein, and tyrosol (**42**–**44**, Figure 1, Table 1) were isolated as phytotoxins from *Biscogniauxia rosacearum* isolated from oak trees in Zagros forests of Gilan-e Gharb, Kermanshah Province, Iran. A strain of the same fungus was recognized for the first time as a pathogen involved in grapevine trunk diseases in Paveh (west of Iran) vineyards, and produced meso-2,3-butanediol as the only phytotoxin. When all the metabolites were tested for phytotoxic activity by leaf puncture assays on *Quercus* ilex L. and *Hedera* helix L., and by leaf absorption assays on grapevines (*Vitis vinifera* L.), at a concentration of 5 × 10^−3^ and 1 × 10^−3^, meso-2,3-butanediol and (3*R*)-5-methyl-6-methoxymellein (**42**) were the most toxic compounds. Nectriapyrone (**19**) and tyrosol (**44**) showed severe necrosis at the highest concentration on *Q. ilex*, while none of the compounds were active on *H. helix*. Furthermore, the phytotoxicity of compounds **42** and **43** was also compared with that of some related natural melleins, namely (3*R*)-mellein (**39**), (*3R*,*4R*)-4-hydroxymellein (**40**), (*3R*,*4S*)-4-hydroxymellein (**41**), (3*R*)-6-hydroxymellein (**45** Figure 1), and (3*R*)-6-methoxymellein (**46**, Figure 1) with a structure–activity relationship (SAR) study. The results obtained showed that on grapevines *(V. vinifera*, L.*)*, the hydroxy group at C-4 of the pyranone moiety negatively affected the phytotoxicity. Instead, phytotoxicity on the same plant was induced by mellein with the C-6 substitution of the aromatic ring either with a phenolic hydroxy or a methoxy group. Finally, the absence of any substituents on the aromatic ring is an essential feature for the toxicity of *Q. ilex* L., suggesting a different mode of action of the melleins on grapevine and oak leaves [42]. Mellein analogue 42 also showed antibacterial activity [40]. Tyrosol and some of its analogues are well known for their antiatherogenic, cardioprotective, anticancer, neuroprotective, and endocrine effects, as recently extensively reviewed [43]. Higginsianins F-I (**47**–**50**, Figure 1, Table 1), a diterpenoid α-pyridone consisting of an unreported N-hydroxyethyl-3-(5-acetyl-4-hydroxy)-α-pyridone ring, and three diterpenoid pyrones, were isolated from the mycelium of another strain of *Colletotrichum higginsianum*. Higginsianin F (**47**) could induce the formation of necrotic spots on *Chenopodium album* L. leaves at a concentration of 2 μg/μL. All higginsianin (**47**–**50**) were also tested on *Amaranthus retroflexus* L. seed germination and applied at four different doses. Higginsianins **47**, **49,** and **50** significantly inhibited *A. retroflexus* seed germination, and compound **47** showed the most potent phytotoxicity, indicating that the presence of *N*-hydroxyethyl-3-(5-acetyl-4-hydroxy)-α-pyridone ring could enhance activity. Instead, higginsianin G (**49**) did not exhibit inhibitory activity, suggesting that stereochemistry may influence the activity. In addition, the presence of OH-7′, as in higgisianin I, induced an increase in phytotoxic activity [62].

### 2.2. α-Pyrones Produced from Fungi Pathogenic for Forest Plants

**Figure 2:**
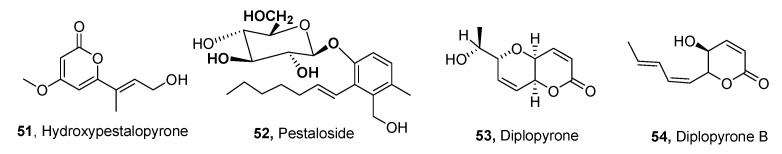
Structures of phytotoxic α-pyrones produced by fungi pathogenic for forest plants.

Pestalopyrone (**19**), hydroxypestalopyrone, and pestaloside (**51** and **52**, Figure 2, Table 2) were isolated from the filamentous fungus *Pestalotiopsis micropspora*, which was found as an endophytic microorganism in the Florida Torreya (*Torreya taxifolia*), whose decline started in the late 1950s and became a rare tree in North America. When assayed at 10 μg using a leaf puncture test on Torreya needles, pestalopyrone, its hydroxy derivative, and pestaloside (**19**, **51**, **52**) induced chlorosis. Compounds **19** and **51** generated symptoms on *Torreya brevifolia* also at a smaller concentration of 1 μg, showing a modest level of host specificity. Pestaloside (**52**) caused identical phytotoxic symptoms on both *T. taxifolia* and *T. breviflia* [63].

(+)-Diplopyrone A (**53**, Figure 2, Table 2), a phytotoxic monosubstituted tetrahydropyranpyran-2-one, was isolated as the main phytotoxin from the liquid culture filtrates of *Diplodia mutila*, a fungal pathogen which causes a form of canker disease on cork oak (*Quercus suber*). *D. mutila* is an endophytic fungus, widespread in Sardinian oak forests. When assayed on the host plants, compound **53** induced necrosis and wilting on cork oak cuttings, while brown discoloration or stewing was observed when tested on tomato [64]. The nonempirical assignment of the absolute configuration (AC) of (+)-diplopyrone was approached by two different methods: (a) the exciton analysis of the circular dichroism (CD) spectrum and (b) the ab initio calculation of the optical rotatory power. Both methods indicate that (+)-diplopyrone is 6-[(1*S*)-1-hydroxyethyl]-2,-4a(*S*),6(*R*),8a(*S*)-tetrahydropyrano[3,2-b]pyran-2-one [65]. Successively, this AC was revised, adopting a new experimental-computational strategy based on the combination of diverse experimental spectroscopies with quantum-mechanical simulations. In particular, diplopyrone was chosen. The close match between the new experimental and simulated infrared absorption and vibrational circular dichroism spectra, as well as the AC, as reported in Figure 2, was definitely assigned to compound **53** [66].

Diplopyrone B (**54**, Figure 2, Table 2) was isolated together with other metabolites from *Diplodia corticola*, another pathogen which causes serious and negative impacts on oak ecosystems, limiting both the vitality and the productivity of these trees. From the same fungal culture filtrates, we also isolated three lactones and a fatty acid ester, named sapinofuranones C and D, diplobifuranylone C, sphaeropsidins A and C, diplopyrone, diplobifuranylones A and B, diplofuranone A, and the (*S*,*S*)-enantiomer of sapinofuranone B. All the metabolites isolated were tested at 1 mg/mL on leaves of cork oak, grapevine cv., ‘Cannonau’, and tomato using the leaf puncture assay. They were also tested on tomato cuttings at 0.2, 0.1, and 0.05 mg/mL. Each compound was tested for zootoxic activity on *Artemia salina* L. larvae. The efficacy of sapinofuranone C and diplopyrone B on three plant pathogens, namely, *Athelia rolfsii*, *Fusarium avenaceum*, and *Phytophthora nicotianae,* was also evaluated. Only diplopyrone B showed strong phytotoxicity inhibition on the vegetative growth of *A. rolfsii* and *P. nicotianae*. All metabolites were inactive in the assay performed for the zootoxic activity, even at the highest concentration used. Diplopyrone B (**54**) also exhibited promising antioomycete activity for the control of *Phytophthora* spp. also considering its lack of zootoxicity [66].

**Table 2:** Phytotoxic α-pyrones produced from fungi pathogenic for forest plants identical data to the entry immediately above).

Compound	Fungal Producer	Other Biological Activities	Ref.
Hydroxypestalopyrone (**51**)	*Pestalotiopsis micropspora*	Not reported	[63]
Pestaloside (**52**)	″	″	″
Diplopyrone (**53**)	*Diplodia mutila*	″	[64]
Diplopyrone B(**54**)	*Diplodia corticola*	Antifungal	[66]

″ It means the species same as above.

### 2.3. α-Pyrones Produced from Fungi Pathogenic for Weed and Parasitic Plants

**Figure 3:**
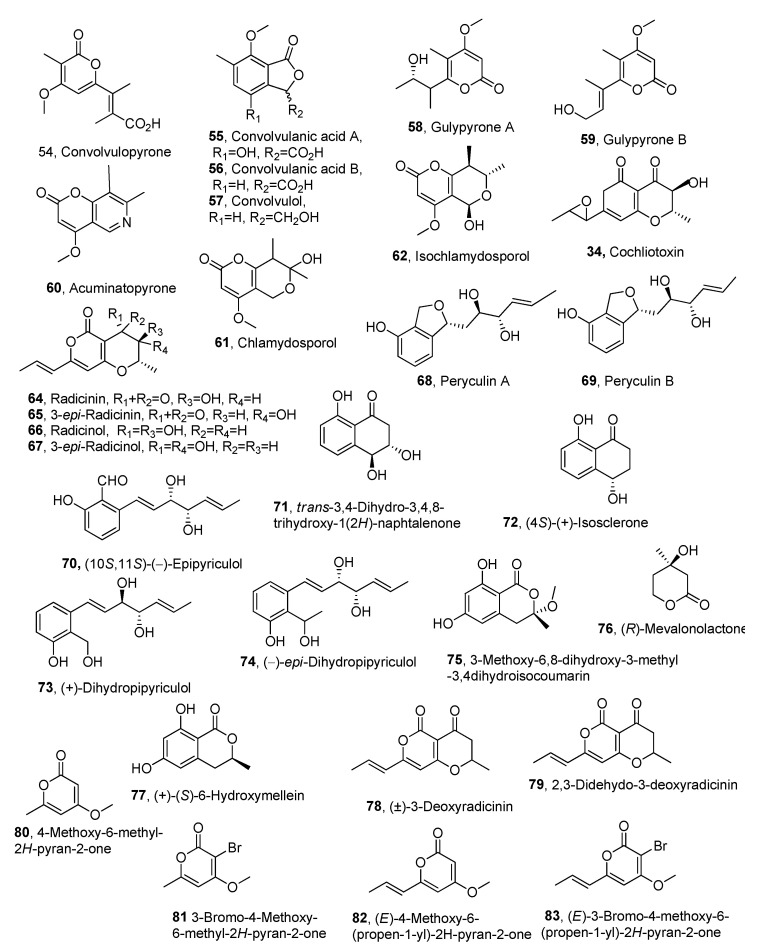
Structures of phytotoxic α-pyrones produced from fungi pathogenic for weeds and parasitic plants.

The phytotoxic convolvulopyrone, convolvulanic acid, convolvulanic acid B, and convolvulol (**54**–**57**, Figure 3, Table 3), were isolated together with ergosterol and ergosterol peroxide from *Phornopsis corzvolvulus* [67]. This fungus is a host-specific pathogen which causes leaf spots and anthracnose lesions in the important perennial weed *Corzvolvulus arvensis* (field bindweed). The necrotic lesions surrounded by yellow haloes are characteristic of phytotoxin production. Bindweed is a severe agricultural constraint around the world, with the exception of tropical regions. Field bindweed infestations have been reported along roadsides, in urban gardens, and in new land and cropland throughout much of Canada [68]. This weed has been classified as one of the most damaging weeds worldwide. Herbicides used to control it have been found to be expensive and ineffective [69]. Strong herbicidal activity against *C. arvernsis* was observed with compounds **55** and **57** at concentrations of 3–5 × 10^−4^ M [67]. The compounds **54**–**57** were tested for their phytotoxic activity on the aquatic plant *Lemna paucicostata,* which showed high sensitivity. The most phytotoxic compound appeared to be convolvulanic acid B (**56**) which induced total inhibition of growth and 100% chlorosis within 12 h at concentrations of 5.9 × 10^−4^ M and within 24 h at concentrations of 3.5 × 10^−4^ M. Metabolites **55** and **57** also inhibited, At concentration of 5.9 × 10^−4^ M, the growth of the same plant around at 80 and 50%, respectively. Metabolite **54** showed very minor phytotoxicity. Tested by leaf puncture assay on bindweed, similar results of phytotoxic activity were observed for each compound [67].

Gulypyrones A and B (**58** and **59**, Figure 3, Table 3), two a-pyrones, were isolated together with phomentrioloxin, phomentrioloxins B and C, 3-nitropropionic, and 4-methylbenzoic acid from a virulent strain of *Diaporthe gulyae* [70]. The fungus was obtained from stem cankers of sunflower and was also known to be a pathogen to saffron thistle (*Carthamus lanatus* L.). *C. lanatus* is a widespread, winter-growing annual weed of both pastures and crops throughout Australia and is considered the most economically important thistle species in New South Wales [71,72]. When assayed at 5 mM on punctured leaf disks of weedy and crop plants 3,-nitropropionic acid, the main metabolite, caused small, but clear, necrotic spots on a number of plant species (*Papaver rhoes*, *Ecballium elaterium*, *Urtica dioica*, *Hedysarum coronarium*, *Mercurialis annua*, *Lactuca serriola*, *Ailanthus altissima*, *and Dittrichia viscosa*). Phomentrioloxin B proved to have a weaker toxicity, causing necrosis on *P. rhoeas and U. dioica*, *M. annua*, *L. serriola*, *A. altissima*, *Picris echioides*, *D. viscosa*, *Helianthus coronarium*, *Helianthus annuus*, and *Aster* sp. Gulypyrone A (**58**), assayed at 5 mM by stem immersion on *Helianthus annuus* plantlets, caused the rapid appearance of clear and very expanded necrosis on leaves, which was associated with the lack of symptoms on the stems. These results suggested that compound **58** can be easily translocated through the vascular system, accumulating in the leaf tissues. 4-Methylbenzoic acid showed a weaker activity, causing small necrosis only to *M. annua*, *U. dioica*, *Solanum nigrum*, and *Aster* sp. All the other compounds were either very weakly toxic or non-toxic. Considering the high toxicity of the culture filtrate, an additive or synergistic activity of all the weakly active metabolites could be hypothesized [70].

Acuminatopyrone, chlamydosporol, and isochlamydosporol (**60**, **61**, and **62**, Figure 3, Table 3) were isolated together with blumenol A, isochlamydosporol, ergosterol, and 4-hydroxybenzaldehyde. *Fusarium tricinctum* is one of the several soil fungi, including other *Fusarium* species, pathogens for seeds of the winter annual grass *Bromus tectorum* (cheatgrass). This weed has become highly invasive in semiarid ecosystems of western North America. However, in these areas, a complete cheatgrass stand failure (‘die-off’), appearing as a natural phenomenon apparently caused by the above-cited complex soil fungi. Testing 4-hydroxybenzaldehyde against *B. tectorum* in a seedling bioassay exhibited the highest phytotoxicity, significantly reducing the coleoptile and radicle length of cheatgrass seedlings. Compound **60** and blumenol showed moderate activity, while compounds **61** and **62** and ergosterol were not significantly different from the control [73].

Cochliotoxin (**63**, Figure 3, Table 3), a dihydropyranpyrone, was isolated together with radicinin, radicinol, and their 3-epimers (**64**–**67**, Figure 3, Table 3) from *Cochliobolus australiensis,* proposed for the biological control of buffelgrass (*Pennisetum ciliare* or *Cenchrus ciliaris*) [73]. The absolute configuration of cochliotoxin was determined using chiroptical Optical Rotatory Dispersion (ORD), Electronic Circular Dichroism (ECD), Vibrational Circular Dichroism (VCD), and computational methods. The same methods were used to confirm that of radicinin, radicinol, and their 3-epimers, previously determined using chemical, spectroscopic, and ECD methods [74]. Buffelgrass is a perennial grass that has become highly invasive in the Sonoran Desert of southern Arizona. All the compounds were tested by leaf puncture bioassay on buffelgrass at the higher concentration (5 × 10^−3^ M); cochliotoxin and 3-*epi*-radicinin (**63** and **65**) were strongly and equally phytotoxic, while radicinin (**64**), compared to them, was less toxic. Radicinol and 3-*epi*-radicinol (**66** and **67**) showed lower phytotoxicity. Compounds **63**–**65** showed high phytotoxicity on tanglehead (*Heteropogon contortus*), but their activity was less than that on buffelgrass, probably as tanglehead is generally less sensitive. Radicinin and 3-*epi*-radicinin (**64** and **65**) were significantly more phytotoxic on Arizona cottontop (*Digitaria californica*) than on buffelgrass and tanglehead, while compounds **66** and **67** were not toxic on tanglehead and Arizona cottontop. Finally, the three most phytotoxic compounds (**63**–**65**) were assayed at the lower concentration (2.5 × 10^−3^ M), and cochliotoxin showed lesser toxicity than either radicinin or 3-*epi*-radicinin on all three grass species used [73]. From the point of view of structure−activity relationships, these results showed that the α,β-unsaturated ketone located between C-4 and C-8 in compounds **63**–**65** should play a significant role in the strong phytotoxic activities of these toxins. The absence of this this moiety in **66** and **67** induced a noteworthy reduction of toxicity. Furthermore, important features involved in modulating phytotoxicity appeared to be the stereochemistry of the chiral C-3 and the presence of the epoxy group in **63**–**65** and **63**, respectively [73]. Radicinin (**64**), already known as a phytotoxin [75,76,77], also showed strong antifungal activity against *Magnaporthe grisea* and *Valsa mali* (IC_50_, the concentration of substance required for 50% inhibition, of 16.3 and 18.2 μg/mL, respectively) [78] as well as strong bacteriocidal activity against *Xylella fastidiosa* [79]. Radicinin (**64**) was isolated as a phytotoxin together with two steroids from the culture filtrates of *Curvularia clavata* when it was obtained by microbiological transformation of progesterone, which was added as substrate when the microorganism reached its exponential growth phase [80].

Successively from the culture filtrate of *Pyricularia grisea*, another pathogen proposed for the biocontrol of buffelgrass, pyriculins A and B (**68** and **69**, Figure 3, Table 3), two monosubstituted hex-4-ene-2,3-diols, together with (10*S*,11*S*)-(−)-epipyriculol, *trans*-3,4-dihydro-3,4,8-trihydroxy-1(2*H*)-napthalenone, and (4*S*)-(+)-isosclerone were isolated (**70**–**72**, Figure 3, Table 3). All the metabolites were bioassayed in a buffelgrass coleoptile and radicle elongation tests, and (10*S*,11*S*)-(−)-epipyriculol proved to be the most toxic compound. Seed germination was much reduced and slowed, and radicles failed to elongate. All five compounds delayed germination, but only (*10S*,11*S*)-(−)-epipyriculol (**70**) prevented radicle development of buffelgrass seedlings, while it had no effect on coleoptile elongation, and the other four compounds caused significantly increased coleoptile development [81]. Furthermore, from the same fungal culture extract (+)-dihydropyriculol, *epi*-dihydropyriculol, 3-methoxy-6,8-dihydroxy-3-methyl-3,4-dihydroisocoumarin, (*R*)-mevalonolactone, and 6-hydroxymellein (**73**–**77**, Figure 3, Table 3) were also isolated [82]. When all the metabolites were bioassayed at 5 × 10^–3^ M on a buffelgrass coleoptile and radicle elongation test, no toxicity was detected. In contrast, (+)-dihydropyriculol and 3-methoxy-6,8-dihydroxy-3-methyl-3,4-dihydroisocoumarin (**73** and **75**) showed a significant stimulation of radical elongation. Moreover, the difference in growth stimulation between compound **73** and its epimer **74** further supported the relationship between absolute configuration and biological activity of these fungal metabolites [82]. Totally, fourteen secondary metabolites were produced by these two pathogens and tested using a leaf puncture assay on the host plant at different concentrations. Radicinin (**64**) and (10*S*, 11*S*)-epipyriculol (**70**) appeared to be the most promising compounds for potential application in agriculture. Thus, their phytotoxicity was also tested on non-host indigenous plants. Radicinin (**64**) showed high target-specific toxicity on buffelgrass, low toxicity to native plants, and no teratogenic, sub-lethal, or lethal effects on zebrafish (*Brachydanio rerio*) embryos. These results prompt the development of a target-specific bioherbicide to be used against buffelgrass in natural systems [83]. These data and the peculiar structural feature of toxin **64** suggested that it be chemically modified to prepare some key hemisynthetic derivatives, and test their phytotoxicity to perform a structure–activity relationship study. In particular, the 3-*O*-*p*-bromobenzoyl, 3-*O*-5-azidopentanoyl, 3-*O*-stearoyl, 3-*O*-mesyl, and 3-*O*-acetyl esters of radicinin were semisynthesized as well as the monoacetyl ester of 3-*epi*-radicinin, the diacetyl esters of radicinol and its 3 epimer, and two diastereomeric hexahydro derivatives in comparison with that of radicinin (**64**). The phytotoxic activity of all derivatives and analogues was assayed by leaf puncture bioassay, and most of the compounds showed phytotoxicity, but none of them had comparable or higher activity than radicinin. Thus, the presence of an α, β-unsaturated carbonyl group at C-4, as well as the presence of a free secondary hydroxyl group at C-3 and the stereochemistry of the same carbon, proved to be the essential feature for activity [84].

Considering these interesting and potential results and a relatively low amount of radicinin produced by fungal fermentation, a novel synthetic strategy to prepare (±)-3-deoxyradicinin (**78**, Figure 3, Table 3) is described. This synthetic method is more efficient than those previously reported in the literature and also shows higher versatility towards the introduction of different side-chains at both C-7 and C-2. Compound **78** showed phytotoxicity against the grass weed buffelgrass comparable to that of the natural phytotoxin radicinin. Therefore, this latter can constitute a more practical synthetic alternative to radicinin as a bioherbicide to biologically control buffelgrass [85].

Radicinin (**64**) and 3-*epi-*radicinol (**64** and **67**) some hemisynthetic radicinin derivative as 3-*O-*acetyl radicinin, 3-*O*-mesyl radicinin, 3-*O*-(5-azidopentanoyl radicinin, 3,4-*O*,*O’*-diacetylradicinol, (±)-3-deoxyradicinin and its synthetic intermediates 2,3-dehydro-3-doxyradicinin 4-methoxy-6-methyl-2*H*-pyran-2-one, 3-bromo-4-methoxy -6-methyl-2*H*-pyran-2-one, (*E*)-4-methoxy-6-(propen-1-yl)-2H-pyran-2-one, and (*E*)-3-bromo-4-methoxy-6-(propen-1-yl)-2*H*-pyran-2 (**79**–**33**, Figure 3, Table 3) were tested for their in vitro anti-cancer activity by MTT assays against three cancer cell models harboring various resistance levels to chemotherapeutic drugs. Radicinin (**64**) showed significant anticancer activity in the micromolar range. The results of the SAR study showed that the lack of activity of radicinol and its 3-epimer (**66** and **67**), and the corresponding 3,4-*O*,*O’*-diacetyl derivative, demonstrated that the carbonyl at C-4 is an important structural feature for anticancer activity, because its presence allows a Michael addition of a nucleophile residue. The activities of (±)-3-deoxy- and 2,3-dehydro-3-deoxy-radicinin (**78** and **79**) were slightly less than that of radicinin, showing that the 3-hydroxy group plays a minor role in the activity. Among the four synthetic, intermediate methoxypyrones (**80**–**83**), only derivative **81** exhibited moderate anticancer activities, while compounds **78**, **82,** and **83** were completely inactive. Interestingly, 4-methoxy-2-pyrone is an important substructural unit of aurovertins B and D, i.e., fungal mycotoxins, showing strong antiproliferative activity against breast cancer cells but with little influence on normal cells [86]. As all four compounds are differently trisubstituted α-pyrones, their difference in activity might be due to the effect of the substituents on the Michael addition of a nucleophilic residue. The presence of the methoxy group at the β-position in all four compounds could reduce their reactivity due to steric hindrance, while the presence of bromine in α-position could instead increase this reactivity; see **81** vs. **80**. The difference between the moderate activity of **81** and the inactivity of **83** may be attributed to the presence of an ethenyl group at the δ-position in the latter, which could strongly reduce its reactivity in the Michael addition. Interestingly, the only active compound (**81**) does not bear a double bond conjugate to the pyrone, which can act as a Michael acceptor. Therefore, it can be hypothesized that its cytotoxicity occurs through a different mechanism with respect to radicinin (**64**) and its derivatives. Not strong variation was observed among the responses of the three different cancer cell lines, suggesting that radicinin (**64**) might exert its anticancer activity through non-apoptotic pathways. Thus, compound **64** could be an interesting candidate to develop further drugs to combat chemoresistant cancers. (±)-3-Deoxyradicinin (**78**), which displays very similar cytotoxicity against the tested tumoral cell lines, may be a more practical alternative to radicinin, as it could be obtained through the novel synthetic strategy, as reported above [87].

**Table 3:** Phytotoxic α-pyrones produced from fungi pathogenic for weeds and parasitic plants: identical data to the entry immediately above).

Compound	Fungal Producer	Other Biological Activities	Ref.
Convolvulopyrone (**54**)	*Phornopsis corzvolvulus*	Not reported	[67]
Convolvulanic acid A (**55**)	″	″	″
Convolvulanic acid B (**56**)	″	″	″
Convolvulol (**57**)	″	″	″
Gulypyrones A (**58**)	*Diaporthe gulyae*	″	[70]
Gulypyrones B (**59**)	″	″	″
Acuminatopyrone (**60**)	*Fusarium tricinctum*		[73]
Chlamydosporol (**61**)	″	″	″
Isochlamydosporol (**62**)	″	″	″
Cochliotoxin (**63**)	*Cochliobolus australiensis*	″	[88]
Radicinin (**64**)	*Cochliobolus australiensis* ″ *Curvularia clavata*	″Antifungal Bacteriocide	[78,88] [79] [80]
3-*epi*-Radicinin (**65**)	*Cochliobolus australiensis*	Not reported	[88]
Radicinol (**66**)	″	″	″
3-*epi*-Radicinol (**67**)	″	″	″
Peryculin A (**68**)	*Perycularia grisea*	″	[81]
Peryculin A (**69**)	″	″	″
(10*S*,11*S*)-(-)-Epipyriculol (**70**)	″	″	″
*trans*-3,4-Dihydro-3,4,8- trihydroxy-1(2*H*)-naphtalenone (**71**)	″	″	″
(4*S*)-(+)-Isosclerone (**72**)	″	″	″
(+)-Dihydropipyriculol (**73**)	″	″	[82]
(-)-*epi*-Dihydropipyriculol (**74**)	″	″	″
3-Methoxy-6,8-dihydroxy-3-methyl -3,4dihydroisocoumarin (**75**)	″	″	″
(*R*)-Mevalonolactone (**76**)	″	″	″
(+)-(*S*)-6-Hydroxymellein (**77**)	″	″	″
(±)-3-Deoxyradicinin (**78**)		Anticancer	[87]
2,3-Dehydro-3-deoxyradicinin (**79**)	″	Not reported	″
4-Methoxy-6-methyl- 2*H*-pyran-2-one (**80**)	″	″	″
3-Bromo-4-Methoxy- 6-methyl-2*H*-pyran-2-one (**81**)	″	″	″
(*E*)-4-Methoxy-6-(propen-1-yl) -2*H*-pyran-2-one (**82**)	″	″	″
(*E*)-3-Bromo-4-methoxy-6- (propen-1-yl)-2*H*-pyran-2-one (**83**)	″	″	″

″ It means the species same as above.

## 3. Conclusions

In conclusion, this review reports the chemical and biological characterization of phytotoxic α-pyrones, comprising those included in very complex structures, produced by fungal pathogens for agrarian, forest, weedy, and parasitic plants. In some cases, the results of SAR study are described as well as the potential practical application of some of these interesting natural compounds and the synthesis of their key derivatives and phytotoxins, also including the a-pyrones treated in this review, are the secondary metabolites produced athogenic fungi as virulence factors and therefore play a fundamental role in the physiological processes that generate the symptoms of the disease. Their isolation and chemical and biological characterization are the first steps to finding environmentally friendly solutions to save agricultural production. The future directions will provide deeper insights into the chemical ecology of these phytotoxic α-pyrones and their potential roles in the development of plant diseases.

## Figures and Tables

**Table 1:** Phytotoxic α-pyrones produced from fungal pathogens for agrarian plants (″: identical data to the entry immediately above).

Compound	Fungal Producer	Other Biological Activities	Ref.
Pyrenocine A (**1**)	*Pyrenoclzaeta terrestris*	Antibiotic activity	[13,15] [18]
Pyrenocine B (**2**)	″	Weak antibiotic activity	″
Pyrenocine C (**3**)	″	″	″
Pyrenochaetic acid A (**4**)	″	Not reported	[17]
Pyrenochaetic acid B (**5**)	″	″	″
Pyrenochaetic acid C (**6**)	″	″	″
Alternaric acid (**7**)	*Alternaria solani*		[19]
10-Deoxyalternaric acid (**8**)	″	″	[20]
10-Deoxy-6,19-dihydro alternaric acid (**9**)	″	″	″
10-Deoxy-6,8,9,19-tetrahydro alternaric acid (**10**)	″	″	″
Solanapyrones A (**11**)	*A. solani* *Ascochyta rabiei*	″ Antibiotic activity	[21,22,23] [24]
Solanapyrones B (**12**)	″	Not reported	[21,22,23]″
Solanapyrones C (**13**)	″	″	″
Solanapyrones D (**14**)	*A. solani*	″	[21,22]
Solanapyrones E (**15**)	″	″	″
Alternasolanol A (**16**)	*Diaporthe angelicae* *Phomopis* sp.	″ Cytotoxic activity	[25] [26]
Alternasolanol J (**17**)	*D. angelica*	Not reported	[25]
Macrosporin (**18**)	″ *Phoma* sp.	″ Antifungal activity	″ [27]
Nectriapyrone (=Pestalopyrone, **19**)	*D. angelica* *Pestalotiopsis oenotherae* *Pestalotiopsis micropspora* *Biscogniauxia rosacearum* *Cosmosporella* sp.	Not Reported ″ ″ ″ Antibiotic	[25] [28] [29] [30] [31]
Foeniculoxin (**20**)	*Diaporthe angelicae*	Not reported	[32]
6-(1-Hydroxypentyl)- 4-methoxy-pyran-2-one (**21**)	*Pestalotiopsis guepinii* *Cosmosporella* sp.	″ Antibiotic	[33] [31]
6-Pentyl-4-methoxy- pyran-2-one (**22**)	*P. guepenii* *Cosmosporella* sp.	Not reported″ Antibiotic	[33] [31]
Ustilaginoiidin A, R_1_=R_2_=H (**23**)	*Villosiclava virens* *Chaetomium* spp.	Not reported Cytotoxic activity	[34] [35]
Ustilaginoiidin B, R_1_=H, R_2_=OH (**24**)	″ *Villosiclava virens*	Not reported	[34]
Ustilaginoidin C, R_1_=R_2_=OH (**25**)	″	″	″
Ustilaginoidin G, R_1_=R_2_=H (**26**)	″	″	″
Ustilaginoidin I, R_1_=H, R_2_=OH (**27**)	″	″	″
Colletopyrandione (**28**)	*Colletotrichum higginsianum*		[36]
Colletochlorin G (**29**)	″	″	″
Colletochlorin H (**30**)	″	″	″
Alternapyrones B (**31**)	*Parastagonospora nodorum*		[37]
Alternapyrones C (**32**)	″	″	″
Alternapyrones D (**33**)	″	″	″
Alternapyrones E (**34**)	″	″	″
Alternapyrones F (**35**)	″	″	″
Ustilopyrones A (**36**)	*Ustilaginoidea virens*	″	[38]
Ustilopyrones B (**37**)	″	″	″
Luteopyroxin (**38**)	*Neofusicoccum luteum*	″	[39]
(*R*)-(−)-Mellein (**39**)	*Neofusicoccum luteum* *Neofusicoccum parvum* *Neofusicoccum australe*	″ Antibacterial, insecticidal, and fungicidal activity	[39] [40]
(3*R*,4*S*)-(−)-4-Hydroxymellein (**40**)	*Neofusicoccum luteum* *Neofusicoccum parvum*	Not reported Antibacterial activity	[39] [40]
(3*R*,4*R*)-(−)-4-Hydroxymellein (**41**)	*Neofusicoccum luteum* *Neofusicoccum parvum*	Not reported Antibacterial activity	[39] Antibacterial activity
(3*R*)-5-Methylmellein (**42**)	*Biscogniauxia rosacearum*	Not reported	[41]
(3*R*)-5-Methyl-6-methoxymellein (**43**)	″	″ Antibacterial activity	″ [40]
Tyrosol (**44**)	″	Not reported Antiatherogenic, cardioprotective, anticancer, neuroprotective, and endocrine effects	[42] [43]
(3*R*)-6-Hydroxymellein (**45**)	″	Not reported	[42]
(3*R*)-6-Methoxymellein (**46**)	″	″	″
Higginsianin F (**47**)	*Colletotrichum higginsianum*		[43]
Higginsianin G (**48**)	″	″	″
Higginsianin H (**48**)	″	″	″
Higginsianin I (**50**)	″	″	″

″ It means the species same as above.

## Data Availability

The data discussed are obtained from the article on the topic trated by SciFinder research.
